# Tenascin-C and mechanotransduction in the development and diseases of cardiovascular system

**DOI:** 10.3389/fphys.2014.00283

**Published:** 2014-07-29

**Authors:** Kyoko Imanaka-Yoshida, Hiroki Aoki

**Affiliations:** ^1^Department of Pathology and Matrix Biology, Mie University Graduate School of MedicineTsu, Japan; ^2^Mie University Research Center for Matrix BiologyTsu, Japan; ^3^Cardiovascular Research Institute, Kurume UniversityKurume, Japan

**Keywords:** extracellular matrix, tenascin-C, matricellular protein, mechanotrasduction, coronary artery, heart, aortic dissection

## Abstract

Living tissue is composed of cells and extracellular matrix (ECM). In the heart and blood vessels, which are constantly subjected to mechanical stress, ECM molecules form well-developed fibrous frameworks to maintain tissue structure. ECM is also important for biological signaling, which influences various cellular functions in embryonic development, and physiological/pathological responses to extrinsic stimuli. Among ECM molecules, increased attention has been focused on matricellular proteins. Matricellular proteins are a growing group of non-structural ECM proteins highly up-regulated at active tissue remodeling, serving as biological mediators. Tenascin-C (TNC) is a typical matricellular protein, which is highly expressed during embryonic development, wound healing, inflammation, and cancer invasion. The expression is tightly regulated, dependent on the microenvironment, including various growth factors, cytokines, and mechanical stress. In the heart, TNC appears in a spatiotemporal-restricted manner during early stages of development, sparsely detected in normal adults, but transiently re-expressed at restricted sites associated with tissue injury and inflammation. Similarly, in the vascular system, TNC is strongly up-regulated during embryonic development and under pathological conditions with an increase in hemodynamic stress. Despite its intriguing expression pattern, cardiovascular system develops normally in TNC knockout mice. However, deletion of TNC causes acute aortic dissection (AAD) under strong mechanical and humoral stress. Accumulating reports suggest that TNC may modulate the inflammatory response and contribute to elasticity of the tissue, so that it may protect cardiovascular tissue from destructive stress responses. TNC may be a key molecule to control cellular activity during development, adaptation, or pathological tissue remodeling.

## Introduction

Living tissue is composed of cells and extracellular matrix (ECM). In the heart and blood vessels, which are constantly subjected to mechanical stress, ECM molecules form well-developed fibrous frameworks to maintain the tissue structure by supporting the shape and position of cells, integrating and transmitting mechanical forces generated inside the cells to whole tissue. ECM is also important for biological signaling, which influences various cellular functions in embryonic development, and physiological/pathological responses to extrinsic stimuli. Tenascin-C (TNC) is a non-structural ECM protein highly expressed in morphogenesis and tissue remodeling, and has a wide range of effects on cell responses. Emerging evidence suggests that TNC may be involved in mechanotransduction in response to mechanical stress. In this review, we will focus on the adaptive role of TNC in the mechanical stress response in the development and pathological state of the cardiovascular system.

## Overview of extracellular matrix in cardiovascular system

### Fibrous extracellular matrix

Of all the organs of the body, the large arteries, particularly the aorta, are subject to the greatest mechanical stress. They have a well-organized fibrous framework. In the tunica media, multilayered elastin sheets (lamellae) connected by fine elastin fibers form a three-dimensional continuous network that links smooth muscle cells. This elastin network of the arterial wall functions as an elastic reservoir protecting the tissue from destructive stress. The outermost layer, the tunica adventitia, consists of a collagen-rich ECM and helps prevent vascular rupture at extremely high pressures (Wagenseil and Mecham, 2009). In the heart, the major structural component of the ECM is collagen, which also forms a three-dimensional network interconnecting myocytes to each other and to the vasculature (Caulfield and Borg, 1979; Borg and Caulfield, 1981). The fibrous skeleton composed of collagen is continuous with the annulus fibrosus cordis, the support apparatus of the tricuspid, mitral, and aortic valves to the cardiac muscle in a manner analogous to the attachment of tendons to skeletal muscle (Hinton and Yutzey, 2011). This stress-tolerant collagenous network not only contributes to passive elastic properties of the heart but also to the transmission of mechanical forces to and from the cardiomyocytes (reviewed in Sussman et al., 2002; Bowers et al., 2010; Borg and Baudino, 2011).

### Non-structural matrix, matricellular protein

In addition to the fibrous ECM, a unique functional category of non-structural ECM, matricellular proteins, are receiving increasing attention (Bornstein, 2009). Matricellular proteins constitute a growing family (Table 1) that originally included thrombosondin-1 (TSP1), SPARC (secreted protein, acid and rich in cysteine; osteonectin), and TNC (Sage and Bornstein, 1991), and then TSP2, osteopontin, CCN1, CTGF (CCN2), and tenascin-X were added (Bornstein and Sage, 2002). They have common unique properties: (1) expressed at high levels during development and in response to injury; (2) do not subserve structural roles but function as modulators of cell-matrix interactions; (3) bind to many cell-surface receptors, other ECM molecules, growth factors, cytokines, and proteases; (4) generally induce de-adhesion, in contrast to the positive adhesivity of most matrix proteins (Bornstein and Sage, 2002). The term has become used more widely and new members, such as galectins and periostin, have joined the group (Bornstein, 2009). In cardiovascular development, significant roles of periostin have been reported (Conway and Molkentin, 2008; Inai et al., 2008; Norris et al., 2008, 2009; Ghatak et al., 2014). It is also noteworthy that some members, such as SPARC, osteopontin, and periostin, have been found to be related to developing bone and teeth, which are subjected to strong mechanical stress.

**Table 1:** **Matricellular proteins**.

Thrombospondins	
TSP-1	
TSP-2	
Secreted protein acidic and rich in cysteine (SPARC/osteonectin)	
Tenascin family	
Tenascin-C	
Tenascin- X	
Osteopontin	
CCN family	
CCN1	Cysteine-rich angiogenic inducer (CYP-61)
CCN2	Connective tissue growth factor (CTGF)
CCN3	Nephroblastoma overexpressed (Nov)
CCN4	Wnt-induced secreted protein-1 (WISP-1)
CCN5	WISP-2 connective tissue growth factor-like protein (CTGF-L)
CCN6	WISP-3
Periostin	
Galectins	
Plasminogen activator inhibitor type 1 (PAI-1)	
Fibulin-5	
Small leucine-rich proteoglycans (Biglycan, Decorin, Lumican, Fibromodulin)	

## Tenascin-C

### The tenascin family

Tenascins are a family of multimeric ECM glycoprotein characterized by an N-terminal globular domain and heptad repeats, which facilitate multimerization; one or more tenascin-type epidermal growth factor (EGF)-like repeats; a series of fibronectin (FN) type III domains, and a C-terminal fibrinogen-related domain. There are six names for the tenascin gene products: tenascin-C, X, R, Y, W, and N (Tucker et al., 2006; Tucker and Chiquet-Ehrismann, 2009). TNC was the first tenascin found to be highly expressed in tendons and embryonic ECM (Chiquet-Ehrismann et al., 1986). It was discovered independently in several laboratories as glioma mesenchymal ECM antigen, myotendinous antigen, cytotactin, and J1 glycoprotein (reviewed in Tucker et al., 2006; Chiquet-Ehrismann and Tucker, 2011). Tenascin-R is the second member and is predominantly expressed in the central and peripheral nervous systems (Rathjen et al., 1991). Tenascin-X is a mammalian tenascin primarily expressed in loose connective tissue such as the dermis, epimysium, and blood vessels (Matsumoto et al., 1992; Bristow et al., 1993) Mutations in tenascin-X can lead to a type of Ehlers–Danlos Syndrome (reviewed in Bristow et al., 2005) Tenascin-Y is an avian tenascin similar to mammalian tenascin-X (Hagios et al., 1996). Tenascin-W (Weber et al., 1998) is found primarily in pre-osteogenic areas, the kidney, smooth muscle, and most prominently also in cancer stroma. Tenascin-N is most recently discovered tenascin and is similar to tenascin-W (Neidhardt et al., 2003).

### Biological role of tenascin-C

TNC is the best characterized member of the family (Orend and Chiquet-Ehrismann, 2006; Midwood and Orend, 2009; Chiquet-Ehrismann and Tucker, 2011; Midwood et al., 2011; Udalova et al., 2011; Brellier and Chiquet-Ehrismann, 2012; Chiquet-Ehrismann et al., 2014) and is a typical matricellular protein. It is a huge molecule of approximately 220–400 kDa as an intact monomer and is assembled as a hexamer. TNC is found in many developing organs of embryos, down-regulated after birth to a few tissues bearing high tensile stress and locations of high cell turnover, but highly up-regulated during injury, inflammation, regeneration, and cancer (Chiquet-Ehrismann et al., 2014). A number of *in vitro* studies suggest that TNC has a wide range of effects on cell adhesion, motility, differentiation, growth control, and ECM organization via multiple cell surface receptors including integrins α9β 1, αvβ 3, and αvβ 6, Toll-like receptor 4 (TLR4) and syndecan-4 (Orend and Chiquet-Ehrismann, 2006; Midwood and Orend, 2009). As in the case of target disruption of several other matricellular protein genes, TNC knockout mice develop normally (Saga et al., 1992; Forsberg et al., 1996). Recent detailed investigations of various disease models using TNC KO have suggested that TNC may promote tissue healing but enhances inflammation and fibrosis (Midwood et al., 2011; Udalova et al., 2011; Brellier and Chiquet-Ehrismann, 2012; Imanaka-Yoshida, 2012; Chiquet-Ehrismann et al., 2014).

During embryogenesis and tissue remodeling, TNC is expressed transiently at specific sites, suggesting that the expression of TNC is tightly regulated dependent on the cell type and tissue microenvironment (Tucker and Chiquet-Ehrismann, 2009). Many different growth factors, such as TGFβ, FGF, PDGF, and proinflammatory cytokines, are able to induce TNC expression (for a review, see Orend and Chiquet-Ehrismann, 2006; Tucker and Chiquet-Ehrismann, 2009).

A variety of signaling pathways and transcription factors are known to stimulate TNC transcription (reviewed in Chiquet-Ehrismann and Tucker, 2011). These include TGF/Smad 3/4 (Jinnin et al., 2004), TLR4/NFkB (Goh et al., 2010), c-Jun/NFkB (Mettouchi et al., 1997), Notch (Sivasankaran et al., 2009), Sox4 (Scharer et al., 2009), PDGF/Ets (Jinnin et al., 2006), and MEF2c with scleraxis (della Gaspera et al., 2009). Conversely, TNC can trigger a variety of signaling pathways via multiple cell surface receptors. Interestingly, it affects some of the same signaling pathways that initially trigger the expression leading to negative or positive feedback loops (Chiquet-Ehrismann and Tucker, 2011). For example, PDGF can induce TNC expression via the phosphoinositide 3-kinase/Akt pathway (Jinnin et al., 2006) and MAPK pathways (Chiquet et al., 2004) and, in turn, TNC enhances PDGF signaling by cross-talk between PDGFR-β and integrin αvβ 3 with activation of focal adhesion kinase and Src tyrosine kinase (Ishigaki et al., 2011). In contrast, a negative feedback loops is created in the case of small GTPase RhoA as discussed in the next section.

### Induction of tenascin-C by mechano-stress

Mechanical stress is also a strong inducer of TNC. Just as one of its original names, “myotendinous antigen,” suggests, TNC is highly expressed at the myotendinous and osteotendinous junctions (Jarvinen et al., 1999, 2000, 2003) at sites subjected to mechanical stress. High expression of TNC is often observed at the branching point of arteries (Mackie et al., 1992), although the expression level of TNC is generally low in adult blood vessels. Based on this distribution of the molecule, the close association of mechanical stress and TNC has been proposed. Supporting this possibility, load-induced bone remodeling or muscle overload up-regulates the expression of TNC (Webb et al., 1997; Fluck et al., 2000; Mikic et al., 2000; Mackey et al., 2011), while immobilizing tendons down-regulates the expression. In culture, various mechanical stresses including stretching (Chiquet et al., 2004), compression (Jagodzinski et al., 2008), and shear stress (Tan et al., 2013), up-regulate TNC synthesis by fibroblasts, chondrocytes, smooth muscle cells, and endothelial cells.

**Figure 1:**
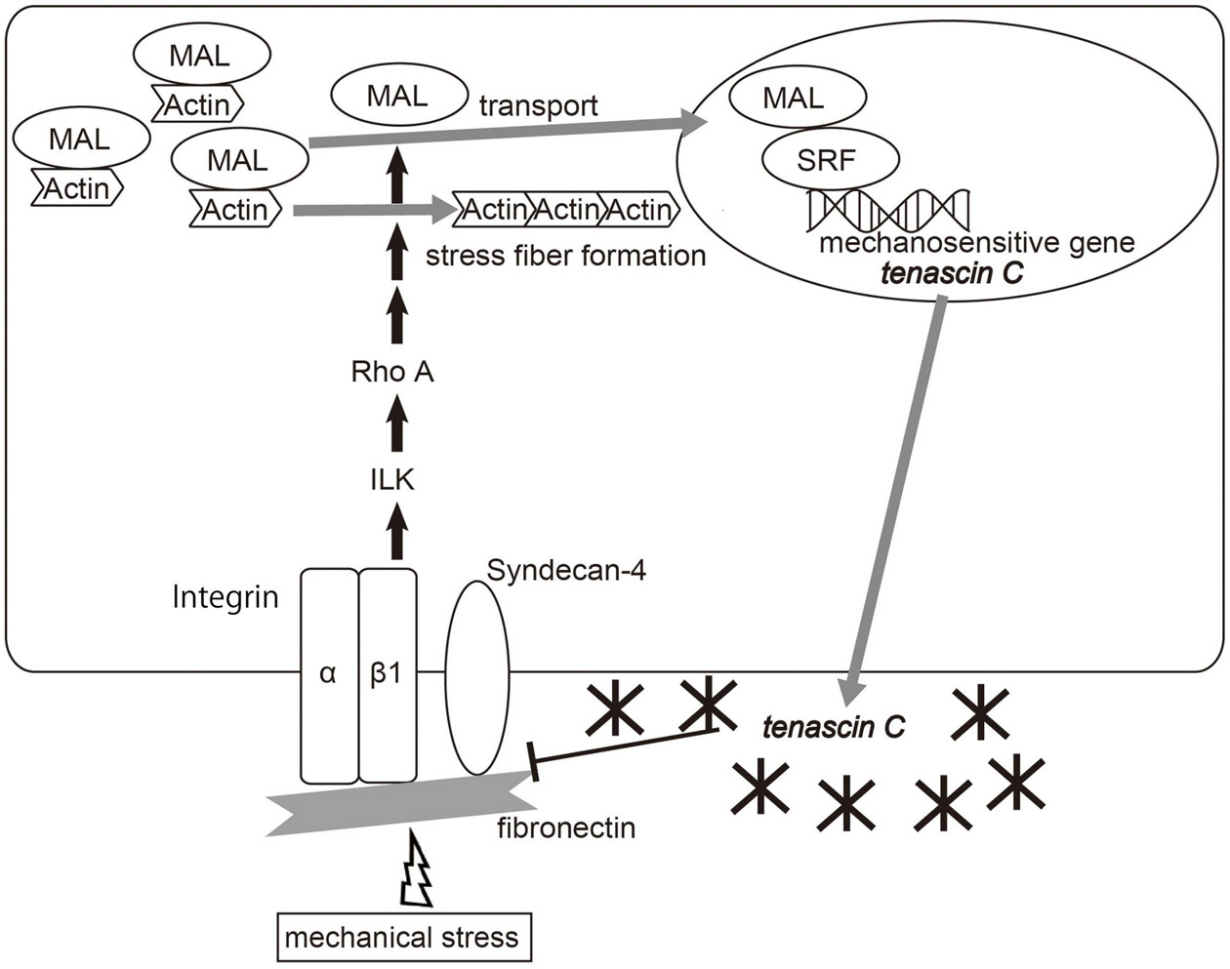
**Diagram of the molecular pathway of the mechano-induction of tenascin-C**. Mechanical strain activates RhoA in fibroblasts, depending on fibronectin, integrin β 1 and integrin-linked kinase (ILK), which causes the reduction of monomeric G-actin by inducing actin assembly and stress fiber formation. Depletion of the G-actin pool frees MAL/myocardin-related transcription factor-A (MRTF-A)/megakaryoblastic leukemia-1 (MKL1) to enter the nucleus, which induces TNC expression partly depending on serum response factor (SRF). Meanwhile, TNC binds fibronectin at the syndecan-4 binding sites and interferes with fibronectin-mediated RhoA activation, and finally suppresses TNC transcription. Adapted from Asparuhova et al. (2009), Imanaka-Yoshida et al. (in press).

Several types of cell-surface proteins, including stretch-sensitive ion channels, are known to sense mechanical forces and translate them into biochemical signals (Kung, 2005). Mechanical inputs can be also detected by mechanosensing apparatus of the focal adhesion complex and transduced to the cytoskeleton (Wang et al., 2009). Chiquet and coworkers have shown a mechanism by which a mechano-signal is transduced at the linkage between the ECM and cytoskeleton, which controls TNC transcription mediated by megakaryoblastic leukemia 1 (MAL or MKL1)/myocardin-related transcription factor A (MRTFA) (Chiquet et al., 2007, 2009; Asparuhova et al., 2009, 2011; Brosig et al., 2010). The cycle stretch of fibroblasts up-regulates TNC transcription, independent of *de novo* protein synthesis, paracrine factors such as TGFβ, and mitogen-activated protein kinases (MAPKs), but depends on actomyosin contractility controlled by the RhoA/ROCK pathway (Sarasa-Renedo et al., 2006) (Figure 1). Mechanical stimuli activate the signaling pathway involving integrin β 1 (Chiquet et al., 2007) and integrin-linked kinase (ILK) (Maier et al., 2008), which induces actin assembly and stress fiber formation via mDia and ROCK (Ridley and Hall, 1992). MAL/MLK1/MRTFA is a coactivator of serum response factor (SRF) and is predominantly localized in the cytoplasm through an interaction with G-actin (Miralles et al., 2003; Guettler et al., 2008). Therefore, depletion of the cytoplasmic G-actin pool following Rho activation causes translocation of MAL into the nucleus, where it induces TNC transcription, partly dependent on SRF (Asparuhova et al., 2011).

RhoA-dependent mechanotransduction requires pericellular fibronectin (Lutz et al., 2010). TNC binds fibronectin at the binding site to syndecan-4, a coreceptor for integrin α5β 1, and has a negative impact on focal adhesion formation and activation of RhoA (Midwood et al., 2006; Lange et al., 2008; Van Obberghen-Schilling et al., 2011). Therefore, mechanically induced TNC may lead to negative feedback from the mechanotrasduction signal. Moreover, since TNC is an elastic molecule that can be stretched to several times its resting length *in vitro* (Oberhauser et al., 1998; Marin et al., 2003), it may contribute to tissue elasticity and protect against mechanical stress.

## Mechanotransduction and tenascin-C in cardiovascular development

During heart development, ECM not only provides structural support for embedded cells but plays an important biological role in the orchestration of cell behavior to form a complex structure with 4 chambers and 4 valves. Accumulated studies have shown diverse functions of various ECM molecules, including hyaluronan, proteoglycans, the collagen family, fibronectin, and periostin (reviewed in Lockhart et al., 2011).

### Heart development and tenascin-C

Specific roles of TNC in heart morphogenesis have long been anticipated based on its strictly regulated temporal expression at specific sites closely associated with cell migration and epithelial-mesenchymal/mesenchymal-epithelial transition: (Wagenseil and Mecham, 2009) differentiation of precardiomyocytes, (Caulfield and Borg, 1979) cushion tissue formation, (Borg and Caulfield, 1981) valve formation, and (Hinton and Yutzey, 2011) coronary vessel formation (Imanaka-Yoshida et al., 2003).

During the development of mouse embryos, the initial expression of TNC is detected in mesodermal cells in the first heart field (FHH), which undergo mesenchymal-epithelial transition and differentiate to cardiomyocytes and endocardial cells. Once the cells differentiate to cardiomyocytes, they rapidly stop expressing TN-C, while endocardial cells continue to express TNC. TNC expression is also detected at the recruitment of precardiac cells from the second heart field (SHF) (Imanaka-Yoshida et al., 2003). Interestingly, cardiomyocytes from the SHF in the outflow tract maintain the expression of TN-C during looping and shortening.

#### Endocardial cushion and tenascin-C

The primitive heart consists of the inner endocardium and outer myocardium and cardiac jelly, composed predominantly of the proteoglycan glycosaminoglycan hyaluronan between the two layers. After cardiac looping, the cardiac jelly expands within the AV canal and outflow tract regions and endocardial cells undergo epithelial–mesenchymal transformation (EMT) and invade it, forming an endocardial cushion (Eisenberg and Markwald, 1995; Person et al., 2005), which is the initial step in valvulogenesis.

A number of reports have demonstrated the expression of TNC in cushion tissue closely associated with EMT of endocardial cells (Hurle et al., 1990; Crossin and Hoffman, 1991; Zhang et al., 1993; Hiltgen et al., 1996; Sugi and Markwald, 1996; Boyer et al., 1999). Indeed, TNC promotes EMT of cancer cells *in vitro* (Nagaharu et al., 2011; Katoh et al., 2013).

Furthermore, Garita et al. have recently reported interesting results suggesting that TNC may provide a structural communication or mechano-communication between the myocardium and endocardium during looping. Using four-dimensional optical coherence tomography (OCT), they found that the endocardium was consistently oriented between the midline of the ventral floor of the foregut and the outer curvature of the myocardial wall throughout the cardiac cycle and that TN-C co-localized with FN at the attachment areas at the outer curvature of the heart wall to the ventral floor of the foregut (Garita et al., 2011).

#### Valve development and tenascin-C

**Figure 2:**
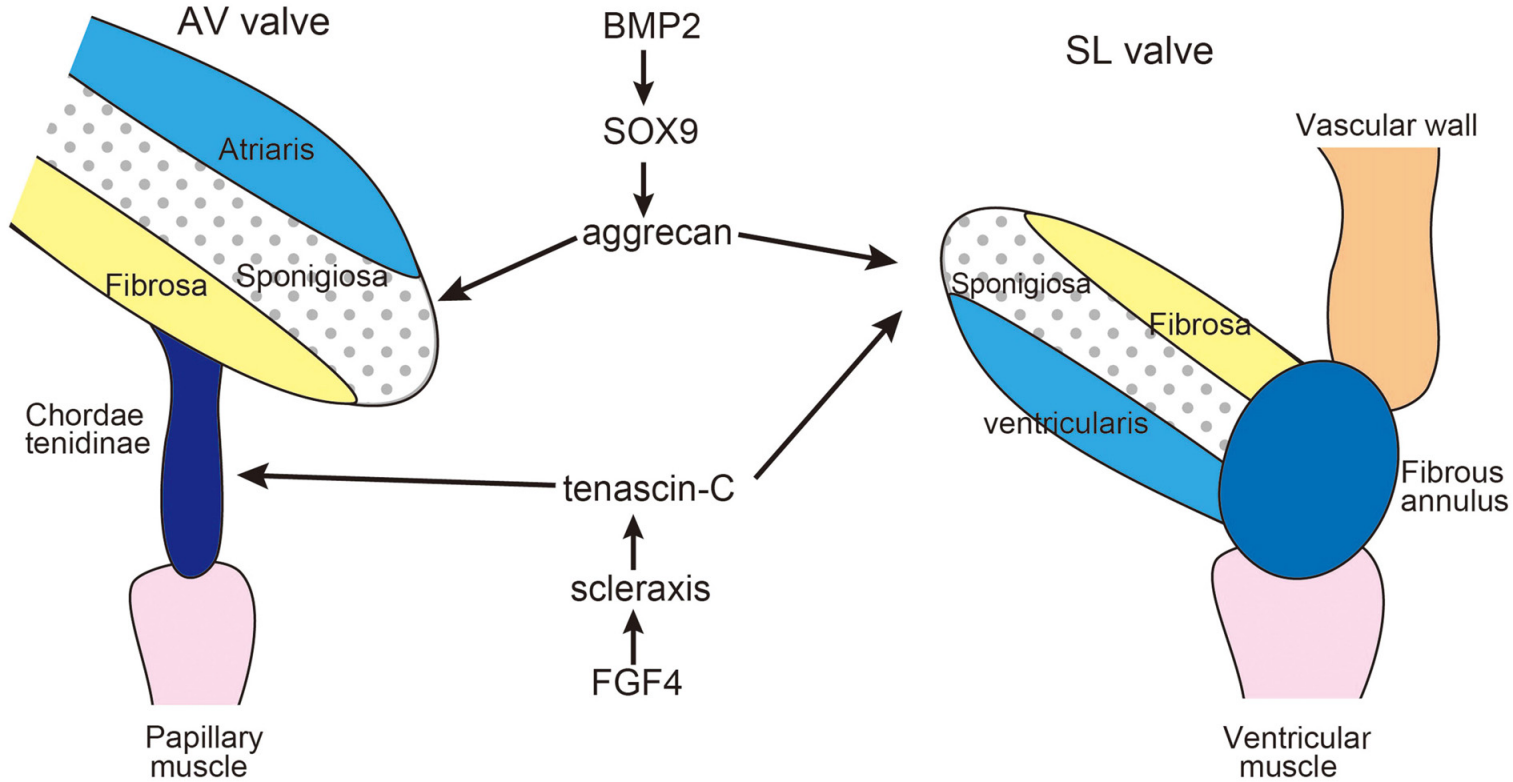
**Diagram of extracellular matrix compartmentalization of the mature AV and SL valves**. During valve maturation, BMP2 signaling induces cartilage-associated genes Sox9 and aggrecan, while FGF4 signaling promotes expression of scleraxis and tenascin, which are characteristic of tendon cell lineages. SL valve precursor cells exhibit both cartilage and tendon-like characteristics. Adapted from Zhao et al. (2007), Hinton and Yutzey (2011).

Later stages of valvulogenesis involve thinning, elongation, and remodeling of the ECM of the primordial valve into three layers: the fibrosa, spongiosa and either the ventricularis of semilunar (SL) valve or the atrialis of the atrioventricular (AV) valve (Lincoln et al., 2004, 2006a; Hinton et al., 2006) (Figure 2). The atrialis/ventricularis are along the flow side of the valves and are rich in elastin fibers. The fibrosa is situated on the ventricular aspect of AV valves and the arterial aspect of the SL valves and is composed of well-organized collagen fibrils. The spongiosa layer of the valve leaflets is rich in chondroitin sulfate proteoglycan, aggrecan, similar to cartilage. The AV valve has supporting structures termed chordae tendinae composed of TNC-rich elastic matrix, which is similar to that of tendons. SL valves lack chordae tendineae, but instead have comparable supporting tissue in the aortic and pulmonic roots and hinge regions (Zhao et al., 2007).

Remodeling of the heart valve primordia shares a regulatory pathway with developing cartilage/tendons (Lincoln et al., 2006b; Hinton and Yutzey, 2011). In the development of limb buds, diversification of cartilage and tendon cells from a common precursor is antagonistically regulated by BMP and FGF signaling pathways. BMP2 not only promotes chondrogenesis but also inhibits tendon development, while FGF4 promotes tendon differentiation (Edom-Vovard et al., 2002; Edom-Vovard and Duprez, 2004).

Similarly, BMP2 signaling activates valve progenitor cells to express Sox9 transcription factor and the aggrecan gene as well as cartilage precursors in limb buds (Lincoln et al., 2006a; Zhao et al., 2007). In contrast, FGF4 signaling activates scleraxis and TNC expression in the valve-supporting apparatus as well as in developing tendons (Lincoln et al., 2006a; Zhao et al., 2007). Hemodynamics is often proposed to be one of the driving forces in valve development (Combs and Yutzey, 2009); however, there is no evidence indicating that mechano-stress might be involved in the induction of TNC during the development of the valves.

### Vascular development and tenascin-C

Another possibility is that TNC may play a role in blood vessel development. In coronary vessels, most vascular progenitors come from the proepicardial organ (PEO) between the primitive heart and the liver bud. Mesenchymal cells from the PEO migrate to the heart and form the epicardium. Epicardial cells undergo EMT, differentiate into endothelial cells and vascular smooth muscle cells (VSMCs), and form a primitive capillary network, which eventually connects to the aorta (see Nakajima and Imanaka-Yoshida, 2013, for review). During this process, TNC is transiently expressed in PEO before cell migration and at epicardial EMT. It is worthy of note that TNC is highly up-regulated and associated with thickening of the vascular wall after the premature vessels are linked with the aorta (Ando et al., 2011), possibly promoting the recruitment of vascular mural cells by facilitating PDGF-BB/PDGFRβ signaling (Ishigaki et al., 2011).

Similar up-regulation of TNC in the vascular wall associated with hemodynamic change is observed during the development of the aorta (Imanaka-Yoshida et al., in press). In E12-13 mouse embryos, weak expression of TNC is detected in the ascending, arch and descending aorta. After ED14-15, when the systemic circulatory system is established, TNC expression is evidently up-regulated and becomes even stronger after birth. In normal adults, the expression of TNC in the aortic wall is generally reduced, although the infra-renal aorta continues to express TNC.

Despite its intriguing expression pattern during cardiovascular development, targeting deletion of the TNC gene causes a grossly normal phenotype (Saga et al., 1992; Forsberg et al., 1996). Our recent preliminary data suggested that over-expression of TNC in the heart may not cause a distinct phenotype, either (unpublished data). Compensatory mechanisms should be present in tissue morphogenesis of the embryo although it is not identified. However, increasing number of studies indicate that TNC is a “stress protein” whose importance becomes apparent when organ homeostasis is challenged by injury or destructive stress such as mechanical overload (Chiquet-Ehrismann et al., 2014), while it is masked during embryonic development.

## Mechanotransduction and tenascin-C in cardiovascular disease

### Mechanotransduction in heart disease

In the heart, extracellular and intercellular mechanical loads are linked to the myofibrils in cardiomyocytes via various mechanosensing complexes (McCain and Parker, 2011). Cadherins links with myofibrils of neighboring cells at intercalated disks, while integrins attach Z-discs laterally to the connective tissue at costameres (Pardo et al., 1983a,b). Costameres are structures related to the focal adhesion complex and critical cytoskeletal elements involved in environmental mechanochemical signal transduction into cardiomyocytes (Samarel, 2005; Russell et al., 2010). They are also the sites where contractile forces generated within cardiomyocytes are transmitted to the surrounding interstitial collagen network (Danowski et al., 1992; Imanaka-Yoshida et al., 1996, 1999, 2004). Costameres may correspond to the myotendinous junction in the sense of transmitting contraction forces of muscle to connective tissue.

Although TNC is not detected in the normal myocardium, it transiently appears upon tissue injury and inflammation in various heart disease (Imanaka-Yoshida, 2012; Okamoto and Imanaka-Yoshida, 2012).

In an acute myocardial infarction model animal, TNC is exclusively localized at the border zone between the intact and infarcted lesion, the most active site of tissue remodeling (Imanaka-Yoshida et al., 2001; Nishioka et al., 2010). As a typical matricellular protein, TNC could loosen the strong costameric adhesion (Imanaka-Yoshida et al., 2001). This “de-adhesion” function may be useful to release surviving cardiomyocytes to reorganize their shape and arrangement; on the other hand, it should reduce the efficiency of the transduction of contraction force of cardiomyocytes. Furthermore, the border zone should be sites subjected to strong stress due to the difference in the physical property of the intact myocardium and necrotic tissue. By exploiting its elastic properties (Oberhauser et al., 1998; Marin et al., 2003), as discussed in the previous section TNC may protect surviving cardiomyocytes in the border zone as a shock absorber. However, there is no formal proof of this concept. In fact, deletion of TNC attenuates adverse ventricular remodeling and improves cardiac function after myocardial infarction in model mice (Nishioka et al., 2010). Therefore, the adaptive role of TNC in heart tissue remodeling has remained elusive.

### Mechanotransduction and tenascin-C in aortic disease

Recently, we found that TNC plays an adaptive role in maintaining the tissue strength of the aorta upon hemodynamic and humoral stress and protects aortic tissue from destructive events (Kimura et al., 2014). In this section we summarize our findings and propose the logic of a maintenance mechanism of tissue strength involving TNC. The aorta must maintain tensile strength to tolerate blood pressure, and must also maintain mechanical flexibility and elasticity to accommodate the stroke volume during the systolic phase and to keep the blood flowing during the diastolic phase. Because the blood pressure and stroke volume fluctuate during the cardiac cycle, circadian rhythm, and depending on physical and mental activities, aortic tissue must have a mechanism that locally optimizes these mechanical properties to meet the changes in hemodynamic demands. The failure of such a mechanism would lead to a mismatch between the mechanical properties and hemodynamic demands, causing central arterial hypertension in the case of excessive aortic stiffness (Agabiti-Rosei et al., 2007) or destructive aortic tissue remodeling including aortic aneurysm and aortic dissection (Cronenwett and Johnston, 2010). Because the mechanical properties of aortic tissue are determined mainly by the composition and architecture of ECM (Cronenwett and Johnston, 2010), the maintenance mechanism of aortic mechanical properties is expected to be tightly coupled with the ECM metabolism. TNC is one of the candidate molecules to maintain the strength of the tissue against mechanical stress.

#### Acute aortic dissection

Acute aortic dissection (AAD) is a medical emergency and the most common aortic disease that is life-threatening (Cronenwett and Johnston, 2010). Patients usually experience the sudden onset of chest or back pain that typically migrates along with the progression of the tearing of the aortic wall. Because patients experience no preceding symptoms, the exact sequence of the events during AAD onset is unknown. However, it is generally accepted that AAD starts with the tearing of the intimomedial layer of the aortic wall, followed by circumferential and longitudinal tearing of the aortic medial wall due to blood rushing into the pseudolumen that is formed between the inner and outer layers of the torn medial layer of the aortic wall. Several genetic disorders are known to predispose the suffering individuals to AAD, including Marfan syndrome, Loeys-Dietz syndrome, vascular Ehlers-Danlos syndrome, bicuspid aortic valve, Turner syndrome, and familial thoracic aortic aneurysm and dissection. However, these genetic disorders account for up to 10% of AAD cases (Cronenwett and Johnston, 2010) and little is known about the etiology of other cases. In addition, the molecular pathogenesis of AAD is largely unknown, partly because animal models that recapitulate the pathological features of human AAD are not available, except for those that are models of genetic disorders.

#### Aortic stress model in mice

**Figure 3:**
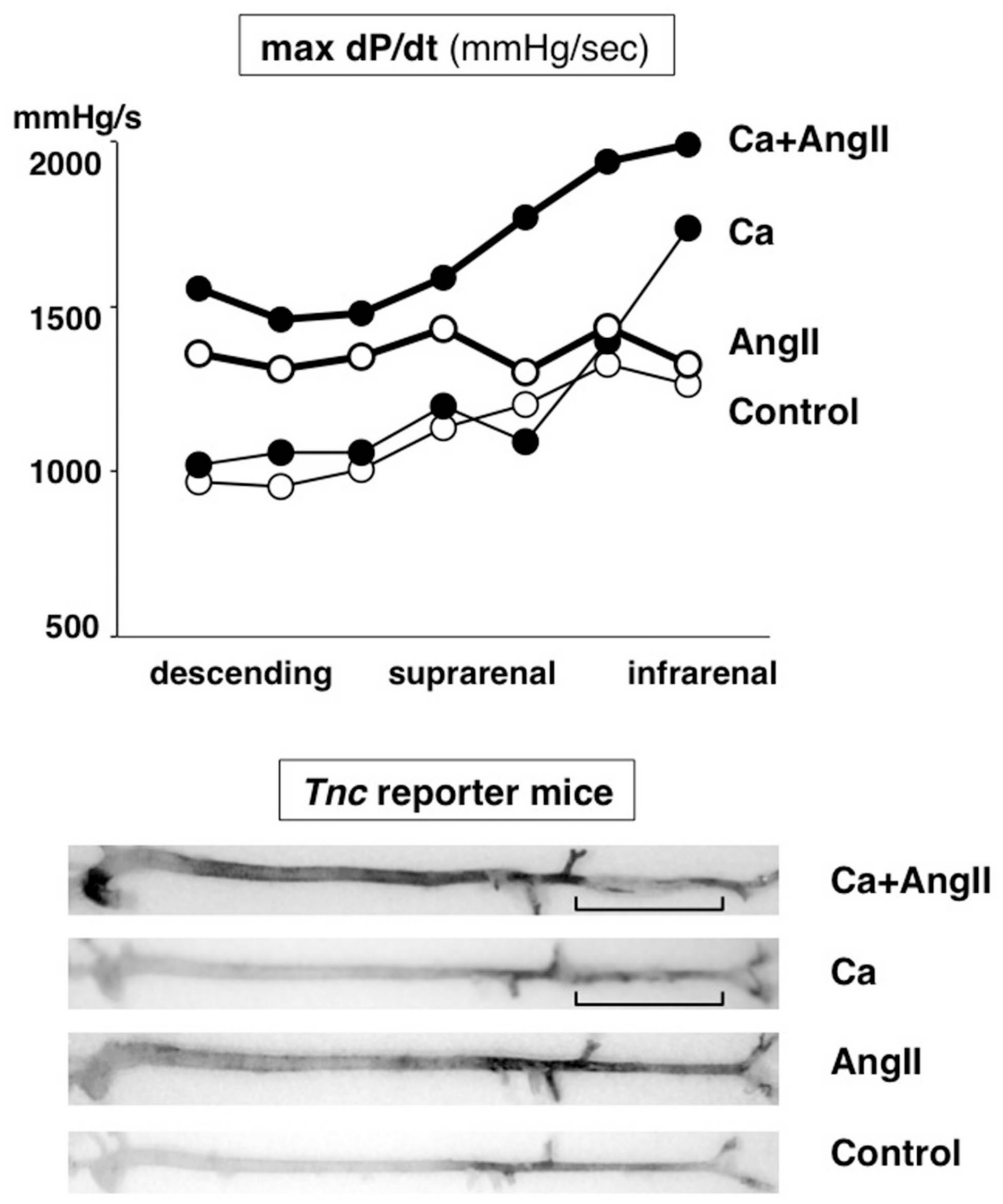
**Mouse model of aortic stress**. Mouse model of aortic stress was created by inducing hypertension with angiotensin II infusion and aortic stiffness with periaortic application of CaCl_2_ in the infrarenal aorta (brackets). Top panel: Hemodynamic stress on aortic wall was evaluated by measuring dP/dt (mmHg/s) with aortic catheterization. Thick lines indicate AngII-treated groups. Closed circles indicate Ca-treated groups. Bottom panel: Stress response of aortic wall was evaluated by X-gal staining of the aorta from *Tnc* reporter mouse, in which blue staining indicates *Tnc* gene activity. Adapted from Kimura et al. (2014).

During the investigation into the pathophysiological role of TNC in the aorta under mechanical and humoral stress, we discovered that deletion of TNC renders mice susceptible to AAD (Kimura et al., 2014). We created a mouse model of aortic stress by inducing aortic stiffness and hypertension (Figure 3), known risk factors for AAD (Jondeau et al., 1999). Aortic stiffness was induced by periaortic treatment of the infrarenal aorta by 0.5 M CaCl_2_, which causes disruption of the elastic lamellae and strong periaortic fibrosis (Ca treatment). Hypertension was induced by continuous infusion of angiotensin II (1 μg/kg/min; AngII treatment), which is known to induce constriction and a proinflammatory response in the vasculature.

The increase in stress in this model was verified by the direct measurement of aortic pressure waves with catheterization. Ca treatment caused an increase in the maximal dP/dt of the distal aorta, while AngII infusion increased that of the proximal aorta. The combination of Ca and AngII treatments (Ca+AngII) increased the dP/dt throughout the aorta. The expression of TNC, as monitored in TNC reporter mice into which the *lacZ* gene was introduced into one of the *Tnc* loci, was observed exclusively in medial smooth muscle cells and faithfully followed the increase in dP/dt.

#### Acute aortic dissection in mice

**Figure 4:**
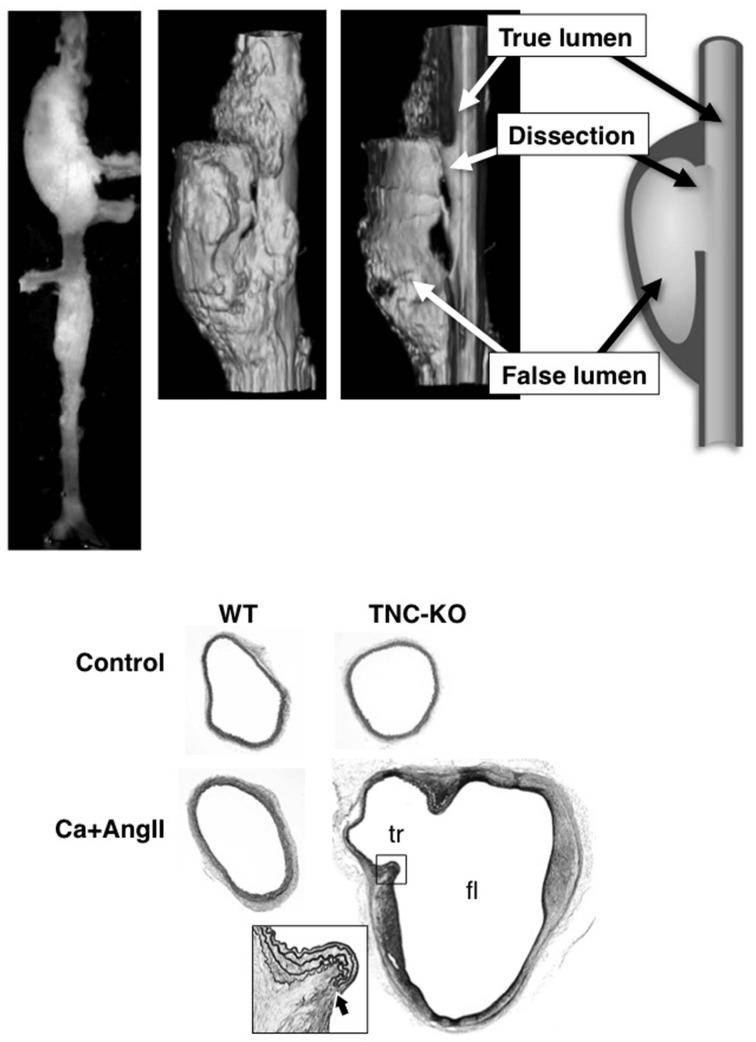
**AAD in TNC-KO mice**. Morphology of mouse AAD in TNC-KO mice. Top panels: A macroscopic image, 3D-reconstitution of optical sections obtained by optical coherence tomography with a cut-out view, and a schematic of the cut-out view. Bottom panels: Elastica van Gieson staining of the suprarenal aorta. True (tr) and false (fl) lumens are indicated in TNC-KO with Ca+AngII treatment. The inset is the magnified view of the dissection site (thick arrow) as indicated by the rectangle. Adapted from Kimura et al. (2014).

To understand the function of TNC in this aortic stress model, we applied Ca+AngII treatment to TNC knockout mice (TNC-KO). Remarkably, only TNC-KO mice developed AAD in the suprarenal aorta (Figure 4), while WT mice showed only aortic wall thickening in the same region of the aorta. Treatment with Ca alone or AngII alone did not induce AAD in either WT or TNC-KO mice. It should be noted that AAD developed in the suprarenal aorta, which is distant from the Ca-treated infrarenal aorta, and in almost all of the cases of AAD we observed a normal-looking segment of the aorta in between. This observation indicated that direct propagation of the inflammation from the Ca-treated infrarenal aorta cannot explain AAD development in the suprarenal aorta. The finding that Ca+AngII treatment greatly enhanced hemodynamic stress led us to conclude that the augmented hemodynamic stress was at least partly responsible for AAD development in TNC-KO mice.

AAD in TNC-KO mice recapitulated the main features of the human aorta, including disruption of the intimomedial layers with otherwise preserved elastic lamellar architecture, intramural hematoma, and formation of a pseudolumen with a double-barrel appearance. One important feature of human AAD was missing; longitudinal dissection of the medial layer. This is probably because the medial layer of the human aortic wall consists of about a 100 layers of elastic lamellae, while that of the mouse aortic wall consists of only 4–7 layers. Therefore, disruption of only a few elastic lamellae would result in complete disruption of the intimomedial layers, leaving only adventitia.

Transcriptome analysis before AAD development revealed the impaired induction of ECM protein genes and exaggerated the induction of proinflammatory genes in the suprarenal aorta of TNC-KO compared to WT (Kimura et al., 2014). Measurement of the tensile strength of the suprarenal aorta in WT showed a transient reduction 1 week after Ca+AngII treatment, which recovered 6 weeks after Ca+AngII, probably due to the induction of ECM proteins. In contrast, the strength of the suprarenal aorta of TNC-KO mice showed more marked weakening 1 week after Ca+AngII treatment, likely reflecting the impaired induction of ECM proteins. Thus, deletion of the *Tnc* gene and the resultant impairment of ECM gene induction showed a significant impact on the adaptive response in reinforcing tissue strength against the increase in hemodynamic stress.

The exaggerated induction of proinflammatory genes in the TNC-KO aorta may also have a significant impact on the homeostasis of aortic tissue. Indeed, imaging cytometric analysis of the TNC-KO aorta showed much more infiltration of CD45-positive inflammatory cells that showed stronger activation of NFκB and STAT3 compared to the WT aorta before AAD development, probably reflecting the proinflammatory environment in the TNC-KO aorta. Interestingly, activation of SMAD2, a downstream molecule of TGFβ signaling, was reduced in VSMCs in the TNC-KO aorta, concomitant with the reduction in the expression of smooth muscle α-actin, indicative of compromised VSMC differentiation. Impaired TGFβ signaling may explain the impairment of both the differentiation of VSMCs and induction of ECM genes, because TGFβ is a strong inducer of VSMC differentiation (Kumar and Owens, 2003) and a master regulator of ECM genes (Bobik, 2006). Consistently, TGFβ is reported to protect the aorta from rupture by angiotensin II infusion in ApoE-deficient mice (Wang et al., 2010), possibly by stabilizing the inflamed aortic tissue (Dai et al., 2005), in contrast to its pathogenic role in Marfan syndrome (Dietz, 2010). Modulation of the cytokine environment may explain the marked reduction in the tensile strength of the aorta and AAD development upon aortic stress by Ca+AngII treatment in TNC-KO mice, although exactly how TNC modulates the cytokine environment remains to be elucidated.

#### Role of tenascin-C in the protection of aortic tissue

**Figure 5:**
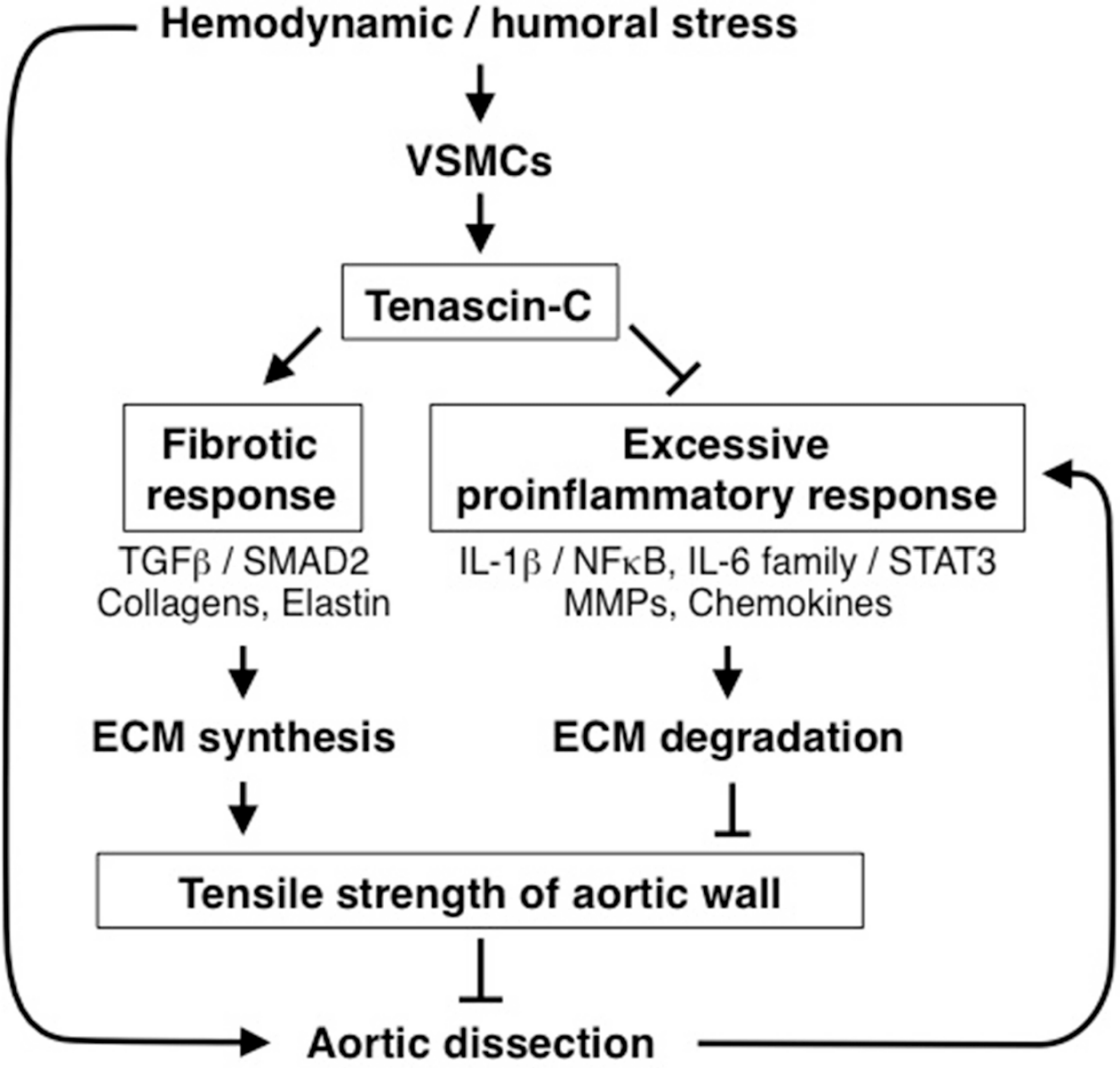
**Role of TNC in protection of aortic tissue**. Diagram of the role of TNC in the stress response of aortic tissue. Hemodynamic and humoral stress induces TNC expression by vascular smooth muscle cells (VSMCs). TNC, in turn, maintains the fibrotic response and ameliorates the excessive proinflammatory response to reinforce the tensile strength of the aortic wall, thus preventing AAD development.

From the viewpoint of aortic homeostasis and AAD pathogenesis, TNC can be regarded as a stress-activated molecular damper (Figure 5); it is inactive under normal conditions, but once the tissue experiences high mechanical stress it is activated and works to reinforce tissue strength by inducing ECM proteins and at the same time by ameliorating the excessive proinflammatory response. These findings may be clinically relevant, because elevation of tissue and serum TNC levels has been reported in both Stanford type A and type B human AAD (Nozato et al., 2013; Trescher et al., 2013). It is also noteworthy that in TNC-KO mice, aortic wall stiffness was increased only in the infrarenal abdominal aorta where TNC was expressed at a low level (our unpublished data). This suggests that TNC may also participate in the maintenance of the flexibility of aortic walls in certain situations.

## Adaptive role of tenascin-C in the mechanical stress response

As observed in the aortic stress model discussed above, adaptive or destructive tissue remodeling upon hemodynamic and humoral stress could be associated with the inflammatory response. Indeed, mechanical forces influence the production of inflammatory mediators (Wang and Thampatty, 2008; Yang et al., 2008). Alternatively, strong mechanical stress may cause minimal injury, which would evoke inflammation and secondary matrix synthesis as a repairing response.

Generally, TNC expression is closely associated with tissue injury and inflammation in various pathological states, which makes TNC a hallmark of inflammation for clinical diagnosis (Imanaka-Yoshida, 2012; Okamoto and Imanaka-Yoshida, 2012). In fact, inflammatory cytokines induce TNC. A growing body of evidence suggests that TNC activates TLR4 signaling, leading to greater cytokine secretion and more TNC synthesis, forming a positive feedback loop to augment inflammation (Midwood et al., 2009; Goh et al., 2010). The exaggerated induction of proinflammatory genes in the stressed aorta of TNC-KO seems to be inconsistent with the current consensus. It is well-known that TNC has diverse functions in a context-dependent manner and they are sometimes conflicting. Since TNC can bind various cell-surface receptors, different signals from one molecule may be transduced via different receptors depending on the cell type.

Obviously, TNC is not the only ECM molecule involved in the response to mechanical stress. Mechanical stimuli can generally up-regulate the gene expression, synthesis and organization of various ECM molecules. In particular, several matricellular proteins, including CCN1 (Hanna et al., 2009), CCN2 (CTGF) (Schild and Trueb, 2004; Chaqour et al., 2006; Honjo et al., 2012), osteopontin (Endlich et al., 2002), SPARC (Durvasula and Shankland, 2005), and periostin (Yamashita et al., 2013) are induced by mechanical stimuli depending on actin cytoskeleton via common or different pathways. These matricellular proteins show a similar expression pattern to TNC and could modulate the signal transduction and activity of the cells. Furthermore, some are co-localized with TNC and can cooperate or counterbalance each other. For example, TNC and osteopontin are strongly induced in spastic cerebral arteries in a subarachnoid hemorrhage model and TNC induces vasospasm, which is reversed by osteopontin (Suzuki et al., 2013). Periostin directly binds TNC, promoting the organization of a fibrous matrix (Kii et al., 2010). Complex networks of multiple ECM molecules, including matricellular protein, may regulate the adaptive and plasticity responses of the tissue to mechanical overload.

Despite this potential compensatory mechanism, deletion of TNC causes AAD under strong mechanical and humoral stress, which suggests that TNC could play a critical role in protecting vascular tissue from destructive stress responses.

## Conclusion

TNC may be one of the extracellular key modulators controlling the cellular response to mechanical load during development as well as during adaptation or pathological tissue remodeling.

### Conflict of interest statement

The authors declare that the research was conducted in the absence of any commercial or financial relationships that could be construed as a potential conflict of interest.
